# Predictors of medical student interest and confidence in research during medical school

**Published:** 2018-07-27

**Authors:** Jennifer Klowak, Radwa Elsharawi, Robert Whyte, Andrew Costa, John Riva

**Affiliations:** 1 Department of Pediatrics, McMaster University, Ontario, Canada; 2 Department of Health Research Methods, Evidence and Impact, McMaster University, Ontario, Canada; 3 Department of Anaesthesia, McMaster University, Ontario, Canada; 4 Department of Medicine, McMaster University, Ontario, Canada; 5 Department of Family Medicine, McMaster University, Ontario, Canada

## Abstract

**Background:**

Research education and opportunities are an important part of undergraduate medical education. This study’s objectives were to determine students’ interest in research, student self-rated research skills, and to assess potential predictors of research interest and confidence.

**Methods:**

Stakeholder consultation and literature informed a 13-item cross-sectional survey. In 2014, all students enrolled in McMaster University’s School of Medicine in Ontario, Canada were sent an electronic survey and two subsequent reminder e-mails.

**Results:**

The response rate was 81% (498 of 618). Most (n=445, 89%) had prior research experiences. The majority of students (n=383, 86%) wanted more research education and opportunities. Higher rating of their supervisors’ understanding of research was associated with greater interest in research (OR=2.08; 95% CI=1.27–3.41). Home campus (distributed vs. main) was not a significant predictor of research interest. In our adjusted linear regression model, the most significant predictors of higher self-rated research ability were prior thesis work and other prior research experience.

**Conclusion:**

In a survey of a three-year medical school, medical student interest in further research education and opportunities was high and positively predicted by student-rated supervisors’ understanding of research, but not campus location. This study also identified several predictors of student self-rated research ability.

## Introduction

Research education and opportunities during medical school are regarded as a vital aspect of a well-rounded undergraduate medical education. In principle, such programs teach future physicians the skills required to critically evaluate medical literature, collaborate to further medical knowledge and provide context to the application of research findings to medical practice^1^. The importance of these skills has been recognized by medical educators as well as by accrediting bodies, including the Royal College of Physicians and Surgeons of Canada within the Scholar role in its CanMEDS competencies framework.^2^ Additionally, the Liaison Committee on Medical Education (lcme.org) and its Canadian counterpart, the Committee on Accreditation of Canadian Medical Schools (cacms-cafmc.ca) created standards for research education and participation, specifically related to basic science, translating evidence to clinical practice, and offering students opportunities to partake in research. Despite the perceived importance of these skills, there has been a general decline in the number of physician scientists. This has been partly attributed to a lack of research opportunities at the undergraduate medical level and the inability to train and retain young investigators.^3^

The potential impact of undergraduate medical education on research productivity and attitudes toward future research has been previously reported.^4-7^ Additionally, a 2015 meta-analysis demonstrated a positive association of research participation in medical school with short and long-term research productivity.^8^ Evaluation of a mandatory research rotation at the Duke University and Stanford University Schools of Medicine demonstrated that over two-thirds of students who participated were able to publish at least one peer-reviewed manuscript during medical school.^6^ However, a questionnaire across seven medical schools in the United Kingdom reported that only 15% of respondents had submitted a study for publication due to a lack of opportunities to take part in research.^9^ These perceptions were echoed in a survey of three Canadian medical schools in which respondents reported a dearth of research mentors, too little time, and inadequate training in research methodology.^10^ These findings demonstrate the potential impact of undergraduate medical education on students’ academic pursuits and highlight the large variation that exists among medical schools’ research curricula, resources and opportunities.

Considering the potential impact of research curricula in developing physician researchers, understanding students’ research perspectives, confidence and interest is vital to continued curriculum development. A 2015 review on medical students and scholarly research noted that only seven of the included 20 studies were within a decade of publication.^11^ Our aim in this study was to examine student research interest and participation as well as self-rated research ability among MD students. Furthermore, we endeavoured to investigate potential predictors of student interest and self-rated research ability.

We hypothesized *a priori* that student-rated supervisors’ understanding of research, campus attended, higher year in training, self-rated research knowledge gained in the MD program and prior research experience would be positively associated with student interest in research as well as self-rated research ability. We also hypothesized *a priori* that self-rated research ability and interest in research would show a positive association.

## Methods

### Study setting

The Michael G. DeGroote School of Medicine is a three-year medical school in Ontario, Canada. The school has three campuses. The largest campus is McMaster’s original medical school campus in Hamilton with 147 MD students per year. There is substantial basic science, clinical, and epidemiological research activity within the university and associated academic hospitals. Two distributed campuses are located in the Niagara and Waterloo Regions, each with 28 MD students per year. These two medical campuses are based at Brock University and the University of Waterloo, respectively.

The Michael G. DeGroote School of Medicine is accredited jointly by the Liaison Committee on Medical Education (LCME) from the United States and the Committee on Accreditation of Canadian Medical Schools (CACMS), as is the case with all Canadian medical schools. To ensure equivalent access of students to research opportunities across all sites, a faculty member who focused on creating research opportunity was appointed in each distributed campus. No such role exists in the Hamilton Campus, which has abundant research opportunities. There was no mandatory research project required of medical students. Participation in research was encouraged, including access to travel grants and a leave policy that facilitates student presentation of research at peer-reviewed conferences.

### Study design

This study was a cross-sectional survey of all Michael G. DeGroote School of Medicine students at McMaster University. Prior to creation of the survey, we performed a literature search in Medline including a 2015 review on medical students and scholarly research,^11^ scanned Canadian MD program research education websites by school and consulted with administration and research leads from each of McMaster’s three medical education campuses. We were unable to identify a previously validated scale for assessing medical student research attitudes and opportunities during training. Therefore, with input from epidemiologists and curriculum content experts, we developed a 13-item survey (Appendix A) aimed at understanding current research education and opportunities. The survey was pretested by the design team to confirm it was encompassing and measuring intended aspects related to research education and opportunity. The survey was reviewed and piloted in discussion with faculty prior to administration to clarify wording and to determine the typical time it would take for a medical student to complete. Questions were a combination of multiple choice, 5-point Likert scale and free-text comment responses. Demographic data, past and current research involvement, self-rated research ability, and opinions on research curriculum, research education and opportunities were collected. Terms “research education” and “research curriculum” were broadly conceived, including basic and clinical research education.

Participation was incentivised with nine $100 prepaid credit cards provided by the Michael G. DeGroote School of Medicine and distributed to study participants via a lottery system. The Hamilton Integrated Research Ethics Board exempted our study from formal ethics review as quality assurance/program evaluation.

### Study participants and sampling method

In September 2014, an anonymous electronic survey was distributed to students via email from the school’s Program Manager. SurveyMonkey (www.surveymonkey.com) was used to administer the survey. Students generated a unique identifier to track possible duplicate responses and allow for removal of individual respondent level data if later requested. An invitation to participate and two subsequent reminder e-mails were sent via e-mail from September to December 2014. Participation was voluntary. Consent was obtained electronically. Students included first-, second-, and third-year classes and MD/PhD students from all three campuses.

### Data analysis

IBM SPSS Statistics version 20.0 (Armonk, New York, USA) was used for statistical analysis. We generated frequencies for all quantitative variables. Self-rated research ability was a composite score from the sum of eight Likert scale questions such as “defining a research question” and “analyzing results from research.” Missing data were assumed to be missing completely at random and multiple imputation was used. Little’s Missing Completely at Random test was conducted. The estimates were used in the final analysis.

For our primary objective, we ran a logistic regression to model demographic predictors associated with student interest in research. Predictors were chosen *a priori* using prior literature and expert opinion. Research interest was recoded as binary, creating two categories of interest (agree versus unsure or disagree). Goodness-of-fit statistics were conducted using the Hosmer & Lemeshow Test. Receiver-operating characteristic curves were generated to test for discriminability and outliers were detected through screening deviance residuals for absolute values greater than two.

As a secondary objective, we ran a linear regression model to determine the variables that significantly predicted student self-rated research ability. Linear regression assumptions were tested prior to the analysis using a normal P-P plot, scatterplots and a residual versus fitted values plot. Multicollinearity was investigated using tolerance statistics and variance inflation factors. Leverage and Cook’s distance values were examined to detect the presence of outliers. Predictor variables were considered significant if they had a p-value < .05 in the adjusted model.

## Results

Table 1 describes the demographics for student respondents. Students in the MD/PhD program were excluded from analysis due to the substantial differences in training program and low number of MD/PhD students (n = 9, 1.4% of observations). Overall, 498 of 618 MD students answered the survey for a response rate of 81%. Of the students that responded, 177 (40%) were in their first year, 156 (35%) were in their second year, and 112 (25%) were in third year. In addition, 322 students (71%) were from the largest campus while 132 (30%) were from the distributed campuses. The majority of students had either an undergraduate thesis or a graduate-level thesis while only 53 (11%) had no prior research experience. The mean self-rated research ability, rated out of 40, was 24.8 (7.45), indicating that most students perceived their abilities to be mid-way from “weak” to “strong” on Likert scale responses. Of respondents, 161 (32%) reported they were currently involved in research. Most students reporting current research participation were from the main Hamilton campus (year 1, 70% (19 of 27 projects); year 2, 65% (44 of 68 projects); year 3, 67% (35 of 52 projects)). Lastly, 383 students (86%) agreed or strongly agreed that they had an interest in further research education and opportunities.

**Table 1:** Demographic characteristics of medical students^a^

**Year in Program**, no. (%), n=445		**Response Rate by Year, n/n (%)**
1	177 (40%)	177/205 (86%)
2	156 (35%)	156/208 (75%)
3	112 (25%)	112/205 (55%)
**Campus**, no. (%), n=454		**Response Rate by Campus, n/n (%)**
Hamilton	322 (71%)	322/447 (72%)
Niagara	75 (17%)	75/87 (86%)
Waterloo	57 (13%)	57/84 (68%)
**Prior Health-Related Research Experience**, no. (%), n=498		
None		53 (11%)
Undergraduate Thesis		191 (38%)
Graduate Thesis		74 (15%)
Other		180 (36%)
**Interest in More Research Education and Opportunities**, no. (%), n= 445		
Agree		383 (86%)
Unsure or Disagree		62 (14%)
**Commented on Current Research Involvement**, no. (%), n=498		
Yes		161 (32%)
No		337 (68%)
**Self-Rated Research Ability** (Scale: 8 – 40), mean (SD), n=454		24.8 (7.45)
**Self-Rated Research Knowledge Gained in the MD Program** (Scale: 8 – 40), mean (SD), n=426		26.29 (6.14)
**Student-Rated Supervisors’ Understanding of Research** (Scale: 1 – 5), mean (SD), n=447		2.04 (0.70)

a Data are from 498 medical students at a single medical school surveyed in fall 2014.

Table 2 presents the results from the logistic regression model predicting demographic variables associated with student interest in further research education and opportunities. From this model, only an increased students’ perception of supervisors’ understanding of research was significantly associated with interest in pursuing future research education and opportunities. Specifically, for each one-unit increase on a five point scale, the odds of the student having interest in further research education and opportunities increased 2.1 times (95% CI 1.3–3.4 P = .004). Otherwise, interest in further research education and opportunities did not differ based on self-rated research knowledge acquired during the MD program, self-rated research ability, campus, year or previous health related research experience.

**Table 2:** Logistic regression model of variables predicting medical student interest in research education and opportunities^a^

	Unadjusted Model			Adjusted Model		
Variable	Odds Ratio	95% CI^b^	P Value	Odds Ratio	95% CI	P Value
**Undergraduate Thesis**	2.4	0.6-9.1	.19	2.2	0.5-10.3	.29
**Graduate Thesis**	3.2	0.8-12.0	.09	3.2	0.6-16.3	.17
**Other Research Experience**	1.6	0.5-5.1	.45	1.6	0.4-6.6	.47
**Year Two**	0.8	0.4-1.6	.52	1.1	0.5-2.5	.82
**Year Three**	1.1	0.4-2.8	.85	1.4	0.5-4.0	.54
**Main Campus**	1.5	0.3-6.4	.56	1.4	0.3-7.7	.64
**Self-Rated Research Ability**	1.0	1.0-1.1	.14	1.0	0.9-1.1	.88
**Student-Rated Supervisors’ Understanding of Research**	2.0	1.3-3.2	.003	2.1	1.3-3.4	.004
**Self-Rated Knowledge Gained in the MD Program**	1.0	1.0-1.1	.79	1.0	0.9-1.1	.93

a Data are from 498 medical students at a single medical school surveyed in fall 2014.

b CI indicates confidence interval.

c Regression coefficients were unstandardized.

d Interactions tested were self-rated knowledge gained in MD program with undergraduate thesis, self-rated knowledge gained in MD program with graduate thesis and self-rated knowledge gained in MD program with other research experience.

Predictors of students’ self-rated research ability (rated out of 40) are displayed in Table 3 from our adjusted linear regression model. Any type of previous research experience had a significant impact on the self-rated research ability. Completing an undergraduate thesis increased self-rated research ability by 11.5 points (95% CI 3.9-19.0 P = .003) while other research experience increased self-rated research ability by 10.3 points (95% CI 2.7-17.9 P = 0.008). Completing a graduate thesis had the largest was detected and added to the model. The results also showed a statistically significant negative association between the interaction term (knowledge gained in MD program and graduate thesis) and self-rated research ability (P = .002). Therefore, our final linear regression model identified prior research experience, self-rated research knowledge, campus and year in MD program as well as the interaction between self-rated knowledge gained in MD program and prior graduate thesis as significant predictors of self-rated research ability. Predictors in the final model explained 37.8% of the variability (R^2^ = 0.378). Approximately 8-14% of data corresponding to each variable were missing.

**Table 3:** Linear regression model of variables predicting medical student self-rated research ability^a^

	Unadjusted Model			Adjusted Model with Interaction Terms^d^		
Variable	Regression Coefficient^c^	95% CI^b^	P Value	Beta	95% CI	P Value
**Undergraduate Thesis**	2.23	0.32-4.13	.02	11.47	3.92-19.02	.003
**Graduate Thesis**	9.48	7.47-11.50	< .0001	27.54	18.90-36.17	<.0001
**Other Research Experience**	-3.90	-6.34--1.45	.004	10.34	2.74-17.94	.008
**Increasing Year**	-0.81	-1.90-0.27	.14	-0.84	-1.58--0.11	.02
**Main Campus**	1.57	-0.22-3.37	.09	1.57	0.28-2.87	.02
**Interest in Research Education and Opportunities**	0.60	-1.19-2.40	.47	0.15	-0.53-0.84	.66
**Student-Rated Supervisors’ Understanding of Research**	0.70	-1.07-2.47	.41	-0.36	-1.26-0.54	.43
**Self-Rated Knowledge Gained in the MD Program**	0.11	-0.03-0.25	.11	0.40	0.12-0.68	.006

a Data are from 498 medical students at a single medical school surveyed in fall 2014.

b CI indicates confidence interval.

c Regression coefficients were unstandardized.

d Interactions tested were self-rated knowledge gained in MD program with undergraduate thesis, self-rated knowledge gained in MD program with graduate thesis and self-rated knowledge gained in MD program with other research experience

## Discussion

Our cross-sectional survey of medical students found that interest in additional research was high, with 383 respondents (86%) interested in further research education and opportunities. Our logistic regression model identified that a higher student’s perception of their supervisors’ understanding of research was associated with a higher odds of having an interest in pursuing future research education and opportunities, while self-rated research knowledge acquired during their training, self-rated research ability, campus, year of program and previous research experience were not. The linear regression identified predictors associated with student self-rated research ability, specifically a positive relationship with prior research experience, largest campus location, self-rated research knowledge gained during MD program and a negative association with increasing year of medical studies.

In this study we explored student interest in research and self-rated research ability in a three-year MD program and compare distributed to main campuses. There was a robust overall response rate of 81% from a single institution (498 of 618). The joint accreditation of Canadian medical schools by LCME and CACMS applies the same research standards across North America, offering support for generalizability of our findings because of similar core research curriculum objectives. We used an original survey tool for self-rated research ability because true research ability is difficult to assess and therefore there is no previously demonstrated criterion validity. We did not follow a formal survey validation process and our findings should therefore be considered with caution. The predictors used in our adjusted linear regression model explained only 37.8% of the variability and therefore there are other important variables associated with medical student self-rated research ability.

Our study demonstrates a high interest in research education and opportunities (86%) independent of campuses, years and differing research experience in a three-year medical program. A study of British medical schools reported a similar 86% interest in research opportunity among medical students.^9^ Generally, previous studies have demonstrated that medical students have a positive attitude toward research.^11^ Career progression and competitive residency programs have been shown in a meta-analysis to be main motives behind MD student involvement in research.^8^ Medical student research interest at distributed campuses has not been previously reported and we demonstrated that students are similarly interested independent of campus location. Distributed campuses are becoming more prevalent in undergraduate medical education and may be geographically further from the affiliated university’s network of researchers. Programs might benefit from paying special attention to meeting the research opportunities and resources required for these interested students.

Barriers previously identified to student research in four-year medical programs have been reported as time, paucity of mentors, inadequate training in research methodology, and the perception that students would not receive adequate acknowledgement of work.^10^ Barriers have not been formally studied in a three-year MD program. Building on this understanding of barriers to research, we might explain the observed increased student interest in research with increasing student-rated supervisors’ understanding of research through the existence of a positive research role model relationship or sound education in research methodology from these individuals. In a systematic review of predominately survey data, academic mentorship was reported to positively impact personal development, research productivity and career guidance.^12^ Medical schools looking to foster research interest have a variety of mentoring approaches available, such as a recently validated competency-based research mentor training program,^13^ formalized mentorship program or mentorship “speed dating.”^14^

Research experience prior to medical school and self-rated research knowledge gained during the medical program both had a significant positive impact on students’ self-rated research ability, suggesting that previous research experience forges more research confidence. However, an unexpected finding therefore was a small decrease in student self-rated research ability with progressing year in program. Given that the score is self-rated and not an absolute measure, a possible explanation is that students’ perception of research ability became more stringent as they gained experience throughout medical school. Additionally, this small difference in self-rated research ability may not translate to observable differences in real world practice. Previous graduate degree was a substantial predictor of self-rated research ability. The disparate confidence between students with or without a higher degree (MSc or PhD) may convey different educational needs and independence in performing research. In support of this, previous studies have demonstrated that medical students with a higher degree have more knowledge in research.^8^ Further investigation is required to understand how best to address the needs of both groups during medical school, for example two different streams of research education.

The findings of this study, along with qualitative comment data from the survey, were discussed among the Michael G. DeGroote School of Medicine executive committee. Given the large number of qualitative comments, a secondary thematic analysis was subsequently conducted. A review of comments identified that students suggested more staff, academic credit and a centralized opportunity portal as important research facilitators. Further details are provided in a companion publication. As a result of this dialogue, a multifaceted approach to curriculum improvement was then undertaken. A school-based Research Consortium was created, composed of physician research leads (both MD and MD/PhD) as well as current medical students who are assigned student representative roles as part of committees such as research training development, Medical Student Research Day, medical journal and Research Interest Group leads. To facilitate a quick, easily accessible resource for students, online webpages are being developed within the student online portal to provide guidance and education on research involvement and funding sources. In an effort to increase research opportunities by targeting the connection between undergraduate and postgraduate medicine, a pilot project is underway linking medical students with internal medicine residents. To promote evidence-based decision-making, a teaching session within the professional competencies curriculum is being developed. During this session, students practice utilizing an evidence-to-decision framework. Lastly, students undertaking optional research electives within the MD program will undergo a new process whereby the research objectives are reviewed for feasibility.

### Conclusion

In our survey of a three-year medical school, student interest in further research education and opportunities was high and positively predicted by student-rated supervisors’ understanding of research. Attendance at the main versus distributed campus was not a significant predictor of research interest. We also identified several predictors of student self-rated research ability, the strongest of which was a prior graduate degree. Additionally, we presented the medical school’s modifications and additions to the research curriculum following consideration of our survey results. Further research is required to confirm our findings in other populations and settings.

## Appendix A

Student Research Education & Opportunities Survey

1. **What is your current year in the MD program?**
  Year 1 | Year 2 | Year 3 | MD/PhD | Other (please specify)
2. **What is your home base campus?**
  Waterloo | Niagara | Hamilton
3. **The use of science enables medical progress.**
  Strongly Agree | Agree | Unsure | Disagree | Strongly Disagree | Choose Not To Answer
4. **I feel secure in my understanding of medical articles and basic statistics.**
  Strongly Agree | Agree | Unsure | Disagree | Strongly Disagree | Choose Not To Answer
5. **How would you rate your ability in the following research categories:**
  - Defining a research question
  - Doing a research background literature search
  - Building a research team
  - Designing research
  - Running research
  - Analyzing results from research
  - Disseminating findings from research
  - Completing scholarly publications related to research
    <each item has a separate rating scale in a table>
    (weak) 0 with scale of radio check boxes to 9 (strong) | Choose Not To Answer
6. **Did you have any health-related research experience/training prior to entering the MD program? (Please select all that apply)**
  No research experience | Bachelors level thesis | Masters level thesis
  PhD thesis | Participation in Research | Research Assistant work
  Research Class | Choose Not To Answer | Other: _______________
7. **I am interested in getting more education and opportunities to participate in research as part of the MD program.**
  Strongly Agree | Agree | Unsure | Disagree | Strongly Disagree | Choose Not To Answer
8. **My teachers/supervisors in the MD program appear to have a good understanding of medical articles and basic statistics.**
  Strongly Agree | Agree | Unsure | Disagree | Strongly Disagree | Choose Not To Answer
9. **How would you go about getting more involved in research during the MD program if you were interested?**
  <text box>
10. **So far in the MD program, it has provided me knowledge in these research methods:**
  - Basic science methods
  - Scientific method principles
  - Methods for designing a research question
  - Clinical research methods
  - Qualitative research methods
  - Translational research methods
  - Social Science research methods
  - Quality Improvement methods
  - Other: _____________________
    <each item has a separate Likert scale in a table>
    Strongly Agree | Agree | Unsure | Disagree | Strongly Disagree | Choose Not To Answer
11. **Please list the topics of any research you currently are undertaking:**
  <text box>
12. **What particular classes/units in the MD program have provided you education on research methods concepts and translating research in practice?**
  <text box>
13. **Please share with us any thoughts you had on ways to improve the MD program in providing education on research and facilitating research opportunities:**
  <text box>
